# Structural, Mechanical, Electronic, and Optical Properties of Hydrogen-Storage Magnesium-Based Mg_2_XH_9_ (X = Cs, Rb)

**DOI:** 10.3390/ma18163829

**Published:** 2025-08-15

**Authors:** Wenhui Li, Qun Wei, Jing Luo, Xiaofei Jia, Meiguang Zhang, Xuanmin Zhu

**Affiliations:** 1 School of Physics, Xidian University, Xi’an 710071, China; 2 College of Physics and Optoelectronic Technology, Baoji University of Arts and Sciences, Baoji 721016, China; 3 School of Information, Guizhou University of Finance and Economics, Guiyang 550025, China

**Keywords:** hydrogen-rich metal, hydrogen-storage, first-principles calculations, optical properties, mechanical properties

## Abstract

Metal hydrides are emerging hydrogen-storage materials that have attracted much attention for their stability and practicality. The novel magnesium-based metal hydride Mg_2_CsH_9_ was investigated using the CALYPSO software (version 7.0). First-principles predictive methods were then employed to investigate the structural, mechanical, electronic, optical, and hydrogen-storage properties of Mg_2_CsH_9_ and its alkali metal substitution structure Mg_2_RbH_9_. The negative formation energy, compliance with the Born stability criterion, and absence of imaginary modes in the phonon spectrum collectively confirm the thermodynamic, mechanical, and dynamic stability of Mg_2_XH_9_ (X = Cs, Rb), fulfilling the basic criteria for practical hydrogen-storage applications. Mg_2_RbH_9_ is particularly outstanding in terms of its hydrogen-storage capacity, with a gravimetric capacity of 6.34 wt% and a volumetric capacity as high as 92.70 g H_2_/L, surpassing many conventional materials. The pronounced anisotropic characteristics of both compounds further enhance their practicality and adaptability to complex working conditions. An analysis of Poisson’s ratio revealed that the chemical bonding in both compounds is predominantly ionic. The details of the band structures and density of states indicate that Mg_2_CsH_9_ and Mg_2_RbH_9_ are semiconductors. Their optical properties confirm them as being high-refractive-index materials.

## 1. Introduction

Carbon combustion and inefficient fossil fuel utilization are the primary causes of a ~35% increase in CO_2_ emissions [1]. Consequently, the growing global emphasis on sustainable development has made clean energy a focus of interest in the energy sector [2,3,4,5,6,7]. Hydrides, long regarded as promising energy carriers, have attracted increasing attention for their high calorific value and zero pollutant emissions [8]. Their outstanding degree of applicability and environmental compatibility compared with other proposed fuels, makes them a choice material for energy storage [9]. For stationary power systems and portable devices, hydrides offer significant advantages in terms of safety, environmental performance, and cost-effectiveness [10].

However, hydrogen faces numerous technical challenges to its industrialization as a sustainable energy source. Current extensive research efforts aim to discover novel materials with a high hydrogen capacity and a suitable operating temperature range to meet industrial requirements [11]. The superior safety of metal hydrides with regard to compressed or liquefied hydrogen has been demonstrated in recent years, leading to their inclusion in commercial supply chains. Their practical usability is enhanced by their ability to release hydrogen under ideal conditions of ambient temperature and pressure [6,12]. However, metals, hydrides, hydroxides, and their alloys or composites still exhibit certain drawbacks such as relatively weak resistance to corrosive influences from environmental factors like temperature, oxygen, and electromagnetic radiation. In contrast, oxide compounds demonstrate significantly higher stability when used up to 1000 °C [13]. Considering the various available hydrogen-storage materials, magnesium-based hydrides have unique advantages that make them some of the most promising candidates. Magnesium exhibits a high hydrogen- storage capacity (7.65 wt% gravimetric and 110 g H_2_/L volumetric) and is abundant in the Earth’s crust, promising conditions for cost-effective large-scale applications [14]. Cs atoms, which are also uniquely advantageous in hydrogen-storage material design, are characterized by the largest atomic radius (265 pm). The hydrogen storage capacity of cesium-containing hydrogen storage structures can be significantly enhanced by expanding the lattice spacing and optimizing hydrogen adsorption sites [15]. Similarly, the double-perovskite structure has displayed remarkable hydrogen-storage capability. For example, the hydrogen capacities of Mg_2_FeH_6_ and Be_2_FeH_6_ are 5.43 and 7.9 wt%, respectively, highlighting their potential as hydrogen-storage materials [16]. Similarly, Be_2_CrH_6_ and Be_2_MnH_6_ exhibit 7.9 and 7.6 wt% hydrogen contents, respectively, making them strong candidates for onboard hydrogen-storage in new energy vehicles [17].

To screen for novel materials with high hydrogen density, we increased the number of hydrogen atoms within the magnesium-based perovskite framework. Subsequently, leveraging the large atomic radius of cesium (Cs), we introduced Cs atoms to expand the structural space. Finally, we employed the particle swarm optimization crystal structure analysis software (CALYPSO7.0) [18] to search for Mg_2_CsH_9_-type structures. Density functional theory (DFT) calculations confirmed that both the Mg_2_CsH_9_ structure obtained through structural search and the Mg_2_RbH_9_ structure derived via alkali metal substitution exhibited stability and demonstrated excellent hydrogen storage performance. In addition, optical properties such as transmittance, reflectivity, and absorption are closely related to the electronic structure, hydrogenation state, and kinetic behavior of hydrogen-storage materials. The present study therefore also analyzed the optical properties. In both compounds, the incorporation of specific metal atoms (Cs/Rb) into the Mg-H framework may weaken the strong ionic Mg-H bonds, potentially reducing the desorption energy barrier and lowering the required hydrogen release temperature. This mechanism suggests a potential pathway for addressing current energy storage challenges.

## 2. Computational Details

The variable cellular crystal structure of Mg_2_CsH_9_ was simulated using the CALYPSO code, a widely used tool whose reliability for stable crystal structure prediction has been verified in various systems [19,20]. During the prediction process, the number of individuals in each generation was set to 30, and the initial structure was constructed randomly within the symmetry constraints. A total of 50 generations were set to ensure that the structural evolution met the convergence criteria. The structural, electronic, elastic, dynamic, and optical properties of Mg_2_XH_9_ (X = Cs, Rb) were then predicted using the Vienna Ab initio Simulation Package (VASP5.4.4) [21] based on density functional theory (DFT) [22]. Exchange and correlation effects in electron–electron interactions were described using the generalized gradient approximation (GGA) combined with the Perdew–Burke–Ernzerhof functional (PBE) [23]. A plane-wave cutoff energy of 600 eV was applied, and a 6 × 6 × 6 Monkhorst-Pack k-point grid was used to ensure sufficient convergence of the total energy, with a precision of 1 × 10^−5^ eV [24,25]. All structures were subjected to lattice optimization and fully relaxed throughout the computational process. The phonon dispersion curves were computed using the Phonopy(version 2.12) package, employing a 2 × 2 × 2 supercell to evaluate the dynamic stability of the materials.

## 3. Results and Discussion

### 3.1. Structural Stability and Hydrogen Storage Performance

**Figure 1:**
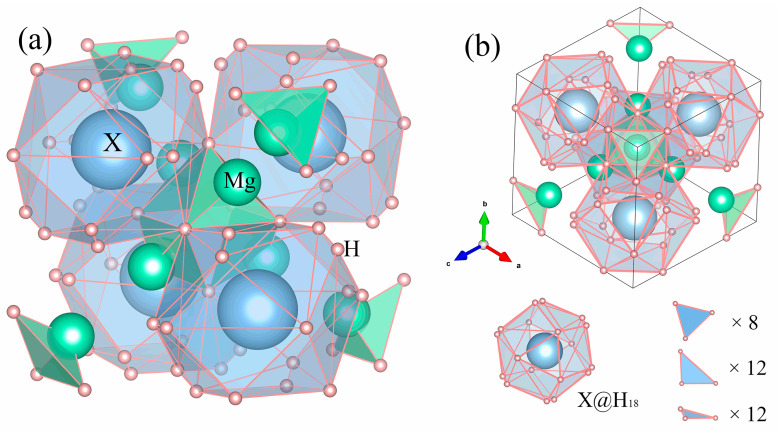
(**a**) Crystal structures of Mg_2_XH_9_ (X = Cs, Rb) compounds and (b) Mg2XH9 (X = Cs, Rb) viewed along the [111] direction.

Mg_2_XH_9_ adopts a cubic structure with space group *Pn*$\bar{3}$ (No. 201). The spatial structure of Mg_2_XH_9_ (X = Cs, Rb) is shown in Figure 1a. Views along the [111] directions are displayed in Figure 1b, where 18 hydrogen atoms form hydrogen cages surrounding the X atom. Specific atomic fractional coordinates are provided in Appendix A. The formation energies of Mg_2_XH_9_ (X = Cs, Rb) were calculated using the following formula [26]:

(1) $${\Delta H}_{f}=\left[E\left({Mg}_{2}XH_{9}\right)-2E\left(Mg\right)-E\left(X\right)-9E\left(H\right)\right]/12$$

where *E*(Mg_2_XH_9_) represents the total energy of Mg_2_XH_9_, and *E*(Mg), *E*(X), and *E*(H) represent, respectively, the average energies of each Mg, X, and H atom in their elemental crystal forms. The formation energies of the two compounds (see Table 1) were negative, confirming their thermodynamic stability.

To further evaluate the thermodynamic stability of these ternary compounds, we searched the Open Quantum Materials Database (OQMD) for relevant stable compounds and constructed decomposition reaction pathways for the Mg_2_XH_9_ structures. The relative formation energy (*E*_f_) was calculated to assess stability: If *E*_f_ < 0, the structure is thermodynamically stable and likely synthesizable via the proposed pathway. If *E*_f_ > 0, the structure has lower thermodynamic stability and risks decomposition. As shown in Table 1, all *E*_f_ values were negative, confirming that these two structures are thermodynamically stable and potentially synthesizable from related compounds.

Mg_2_XH_9_ adopts a cubic crystal structure with three independent elastic constants (*C*_11_, *C*_12_, and *C*_44_) and its mechanical stability was assessed using the Born criteria [27]:

(2) $$C_{11}-C_{12}>0,C_{11}+2C_{12}>0,C_{44}>0$$

As shown in Table 2, both metal hydrides satisfy the stability criteria expressed in Equation (2), confirming their mechanical stability. In cubic crystals, uniaxial compression along the x-axis is represented by *C*_11_, whereas resistance to deformation under pure shear stress is represented by *C*_44_. The *C*_11_ values of both hydrides were higher than their corresponding *C*_44_ values, indicating that their resistance to uniaxial compression was greater than that to shear deformation.

**Figure 2:**
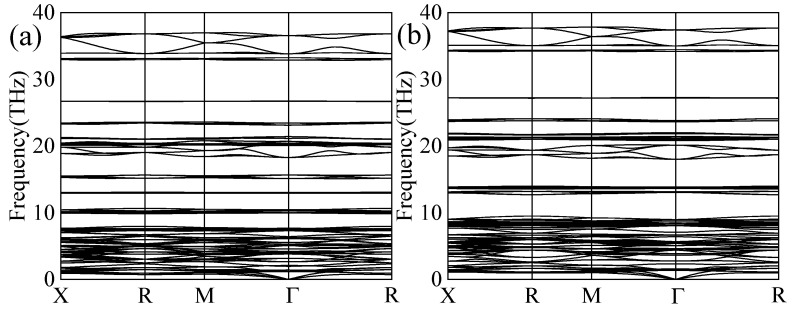
Phonon spectra of (**a**) Mg_2_CsH_9_ and (**b**) Mg_2_RbH_9_.

Phonon dispersion spectra are key indicators of the dynamical stability of materials. As shown in Figure 2, the phonon frequencies of both compounds were non-negative throughout the Brillouin zone. Imaginary frequencies below zero were not observed within the Brillouin zone, thereby confirming the dynamical stability of Mg_2_CsH_9_ and Mg_2_RbH_9_.

To evaluate the hydrogen-storage performance of Mg_2_XH_9_, the gravimetric hydrogen-storage capacity was assessed based on the following formula [28]:

(3) $$C_{wt}\%=\left(\frac{{nm}_{H}}{m_{Host}+{nm}_{H}}\times 100\right)\%$$

where *n* denotes the atomic ratio between hydrogen atoms and the host material (Mg_2_X), and $m_{H}$ and $m_{Host}$ are the molar masses of hydrogen and the host material (Mg_2_X), respectively. The hydrogen-storage capacity of Mg_2_RbH_9_ was 6.34 wt%, demonstrating significant potential compared with common hydrogen-storage materials such as NaAlH_4_ (5.6 wt%) [29] and LaNi_5_H_6_ (1.4 wt%) [30], thus Mg_2_RbH_9_ demonstrates significant potential as a hydrogen-storage material.

Volumetric storage capacity, which quantifies the amount of hydrogen stored within a specific volume, is a key parameter for evaluating the hydrogen storage capability of materials [31]. Its mathematical expression is given as follows:

(4) $$\rho_{vol}=\frac{N_{H}\cdot m_{H}}{V\left(L\right)\cdot N_{A}}$$

where $N_{H}$ denotes the number of absorbed hydrogen atoms, $m_{H}$ represents the molecular weight of hydrogen, *V*(*L*) indicates the absorbent volume (i.e., the cubic unit cell volume in Table 2), and $N_{A}$ is Avogadro’s number. As shown in Table 2, Mg_2_RbH_9_ exhibited a higher hydrogen-storage capacity due to its smaller volume, which is determined by the cation size (Rb^+^). Larger cation sizes lead to increased unit cell volumes, and consequently, a lower volumetric storage capacity.

During desorption, thermodynamic and kinetic factors act synergistically to influence the desorption rate and temperature. The desorption temperature, a key parameter for characterizing hydrogen-storage, is the temperature at which hydrogen becomes released from solid hydrides [32]. Its value is primarily calculated as:

(5) $$T_{des}=\frac{\left|∆H_{f}\right|}{∆S}$$

where ∆*H_f_* (see Table 2) and ∆*S* represent, respectively, the formation enthalpy of solid hydride and the entropy change of hydrogen (approximately −130.7 J/mol) [33]. The ideal temperature range is typically between 233 and 333 K [34]. The calculation results presented in Table 2, show that the desorption temperatures of both Mg_2_CsH_9_ and Mg_2_RbH_9_ fall within this ideal range. The $T_{des}$ values of 285.69 K or 282 K in Table 2 suggest that these structures may release hydrogen at room temperature. Therefore, the materials should be stored in environments slightly below $T_{des}$ or under pressure regulation to suppress hydrogen release. This property makes them suitable for mobile devices requiring rapid low-temperature hydrogen release, avoiding risks associated with high-temperature operations.

### 3.2. Mechanical Properties

The calculation of elastic constants facilitates the assessment of material stiffness, brittleness, ductility, mechanical stability, and elastic anisotropy. In VASP-based calculations, elastic constants are determined using the stress-strain method to comprehensively analyze the mechanical properties of the metal hydrides. Typically, elastic moduli include the bulk modulus, Young’s modulus, shear modulus, and Poisson’s ratio, which are calculated using the following formulas [35,36]:

(6) $$B=\frac{C_{11}+2C_{12}}{3}$$

(7) $$E=\frac{9BG}{3B+G}$$

(8) $$G_{V}=\frac{C_{11}-C_{12}+3C_{44}}{5}$$

(9) $$G_{R}=\frac{5C_{44}\left(C_{11}-C_{12}\right)}{4C_{44}+3\left(C_{11}-C_{12}\right)}$$

(10) $$G=\frac{G_{V}+G_{R}}{2}$$

(11) $$v=\frac{3B-2G}{6B+2G}$$

When investigating mechanical properties, the bulk modulus (*B*), Young’s modulus (*E*), and shear modulus (*G*) characterize a material’s resistance to volumetric compression, elastic deformation, and shear strain, respectively. As shown in Table 3, Mg_2_RbH_9_ exhibits a high resistance to compression. The ductility and brittleness of materials can be assessed using the Pugh ratio (*B*/*G*). When *B*/*G* > 1.75, the material is considered ductile, whereas *B*/*G* < 1.75 indicates brittleness. The calculated *B*/*G* values for Mg_2_CsH_9_ (1.46) and Mg_2_RbH_9_ (1.52) were both below this critical value, confirming their brittle nature. This observation is consistent with the commonly reported low *B*/*G* ratios in metal hydrides [34]. Notably, the brittle characteristics of these compounds have significant engineering relevance in the context of the safe handling and transportation of hydrogen storage systems. Further analysis based on Poisson’s ratio indicates that the ν values of Mg_2_CsH_9_ (0.221) and Mg_2_RbH_9_ (0.23) are both close to 0.25, suggesting that their chemical structures are predominantly governed by ionic bonding. This result agrees well with the theoretical predictions derived from the charge-density distribution calculations.

The elastic anisotropy factor can be expressed using the three independent elastic constants *C*_11_, *C*_12_, and *C*_44_, as defined by the following equation.

(12) $$A=\frac{2C_{44}}{C_{11}-C_{12}}$$

As shown in Table 3, the anisotropy factor of the metal hydrides Mg_2_XH_9_ deviate from 1, indicating elastic anisotropy.

Vickers hardness, melting point, and machinability index ($\mu^{M}$) are key indicators used for assessing mechanical properties, as they not only reflect structural strength but also play decisive roles in practical applicability. To evaluate the mechanical properties, Vickers hardness (based on the Chen model), melting temperature, and machinability index, are estimated using the following formulas [37,38,39]:

(13) $$H_{v}={2\left(k^{2}G\right)}^{0.585}-3$$

(14) $$T_{m}=\left[553+5.911\times C_{12}\right]\pm 300$$

(15) $$\mu^{M}=\frac{B}{C_{44}}$$

The parameter *k* is defined as the ratio of shear modulus to bulk modulus (i.e., *k = G*/*B*). Based on the data in Table 3, the comparable melting points and hardness values of Mg_2_CsH_9_ and Mg_2_RbH_9_ confirm their virtually equivalent crystal structural strength and thermal stability. The machinability index $\mu^{M}$, which correlates with machinability, exhibits a direct proportionality to mechanical processability enhancement [40,41,42,43,44]. As shown in Table 3, it can be reasonably inferred that Mg_2_RbH_9_ shows superior processability compared with Mg_2_CsH_9_.

To further understand the structural stability of the materials, the Debye temperature $\theta_{D}$ was also calculated using the following formula [45]:

(16) $$\theta_{D}=\frac{h}{k}{\left(\frac{3n}{4\pi V}\right)}^{\frac{1}{3}}v_{m}$$

where *h* denotes the Planck constant, *k* is the Boltzmann constant, *n* is the number of atoms per unit cell, *V* is the crystal volume, and $v_{m}$ is the average sound velocity. The Debye temperature generally reflects the highest excited state of the atomic vibrational energy levels in a crystal, and its value is closely associated with the chemical bond strength, lattice dynamic characteristics, and thermal transport properties. A high Debye temperature implies strong interatomic bonding and intense internal interactions within the system [46]. The results in Table 3 suggest that Mg_2_RbH_9_ exhibits tighter internal atomic bonding, demonstrating superior structural cohesion performance.

### 3.3. Electronic Band Structure and Density of States

**Figure 3:**
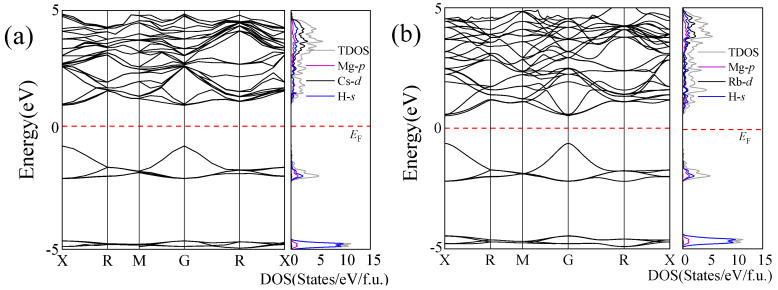
Electronic band structures and density of states of (**a**) Mg_2_CsH_9_ and (**b**) Mg_2_RbH_9_ calculated using the HSE06 method. *E*_F_ denotes the Fermi level.

To investigate the electronic properties of the magnesium-based alkaline metal hydrides Mg_2_XH_9_ (X = Cs, Rb), we calculated their band structures using the GGA functional and the Heyd–Scuseria–Ernzherof (HSE06) hybrid functional in VASP [47,48]. The HSE06 hybrid functional is beneficial for accurately determining the band gap of Mg_2_XH_9_. The calculation results indicate that both materials exhibit direct band gap characteristics, as illustrated in Figure 3. Using the GGA method, the Mg_2_CsH_9_ band gap was calculated to be 0.87 eV, whereas that of Mg_2_RbH_9_ was significantly lower at 0.67 eV. The HSE06 hybrid functional accurately determined the band gap as 1.65 eV (Mg_2_CsH_9_) and 1.12 eV (Mg_2_RbH_9_). The narrowing of the band gap implied a lower electron transition barrier [49], which was identified as a critical factor for enhancing hydrogen-storage performance. This indicates the enhanced potential of Mg_2_RbH_9_ for application as a hydrogen-storage material. Notably, the Fermi level (marked by the red dashed lines) was biased toward the valence band maximum in both cases. Combined with the hole concentration analysis, both materials were identified as p-type semiconductors. This carrier characteristic could potentially optimize the hydrogen-storage performance through a hole-conduction mechanism.

As illustrated in Figure 3, the electronic structure of the Mg_2_XH_9_ (X = Cs, Rb) compounds shows the valence bands being predominantly dominated by the *s*-orbitals of hydrogen atoms, indicating their decisive role in the electronic structure of the valence. The conduction band region is primarily dominated by the d-orbital of the X atoms (Cs/Rb), whereas the *p*-orbital of the Mg atoms participates only marginally in low-energy regions. The highly electronegative X atoms dominate the formation of the conduction band, whereas hydrogen atoms maintain lattice stability through strong ionic bonding.

**Figure 4:**
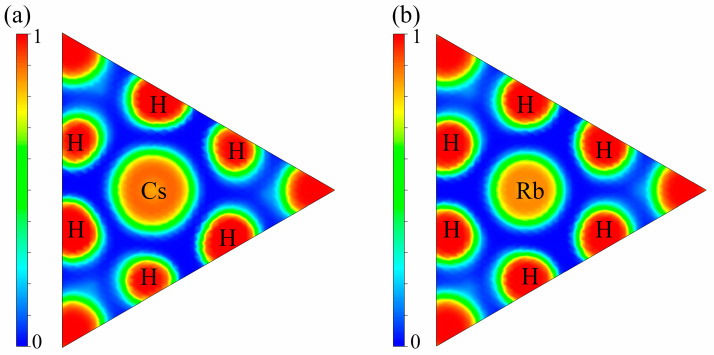
Electronic localization function (ELF) along the [111] direction of (**a**) Mg_2_CsH_9_ and (**b**) Mg_2_RbH_9_.

As illustrated in Figure 4, the electron localization function (ELF) analysis showed that Mg_2_XH_9_ (X = Cs, Rb) exhibits pronounced high electron density regions (ELF ≈ 1) surrounding hydrogen atoms in the (111) crystallographic plane. A pronounced electron localization gradient between the X (Cs/Rb) and H atoms indicates strong localized characteristics in H-X bonding. Bader charge analysis indicates that, on average, each H atom gains approximately 0.473 e from Mg and X atoms (X = Cs) or 0.427 e from Mg and X atoms (X = Rb), which is consistent with the high electron density distribution around H atoms observed in the ELF mapping. The highly localized electron clouds (ELF > 0.8) surrounding the H atoms, combined with significant charge transfer (>0.85 e), collectively demonstrate the ionic hybrid bonding characteristics of the Mg-X-H system. This electronic-scale evidence provides fundamental insights into the structural stability of the materials [50,51].

### 3.4. Optical Properties

The optical properties of Mg_2_XH_9_ (X = Cs, Rb) were comprehensively analyzed and described, focusing on their dielectric function, refractive index, absorption, reflectivity, and energy-loss function.

The dielectric function was calculated as [52,53]:

(17) $$\varepsilon \left(\omega \right)=\varepsilon_{1}\left(\omega \right)+\varepsilon_{2}\left(\omega \right)$$

**Figure 5:**
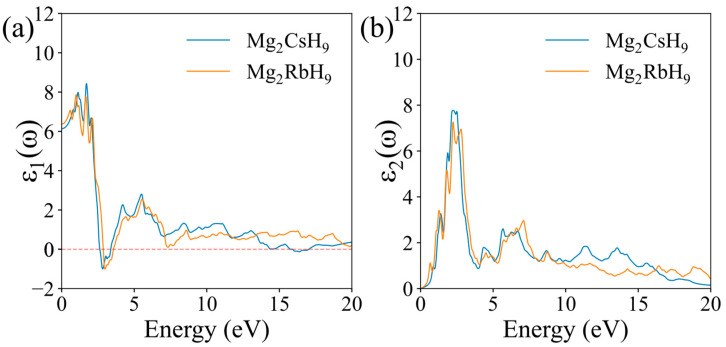
Calculated dielectric functions of Mg_2_XH_9_: (**a**) real part; (**b**) imaginary part.

The real part of the dielectric function $\varepsilon_{1}\left(\omega \right)$, which characterizes the polarizability and dispersion relations in surface electromagnetic responses (Figure 5a), displayed a higher peak value for Mg_2_CsH_9_ (approximately 8.6) compared with Mg_2_RbH_9_ (approximately 7.9) within the low-frequency region (0–10 eV), indicating a stronger polarization response to low-frequency electric fields in Mg_2_CsH_9_. The imaginary part $\varepsilon_{2}\left(\omega \right)$, which reflects the light absorption capacity through electronic band transitions (Figure 5b), displayed peaks around 7.8 and 7.2 for Mg_2_CsH_9_ and Mg_2_RbH_9_, respectively. This confirms the superior low-frequency photon absorption efficiency in Mg_2_CsH_9_, which correlates with the electron transition characteristics of H-X bonds. In the high-energy region (10–20 eV), the real part of the dielectric function $\varepsilon_{1}\left(\omega \right)$ for both materials approached 1 (the vacuum dielectric constant), while the imaginary part $\varepsilon_{2}\left(\omega \right)$ decayed to near zero. This behavior originates from truncation effects in the calculation, which exclude high-energy interband transitions such as inner-shell electronic excitations. In real materials, plasmon resonance (>15 eV) should exist in this energy range, but the independent-particle approximation (IPA) fails to account for such many-body effects.

**Figure 6:**
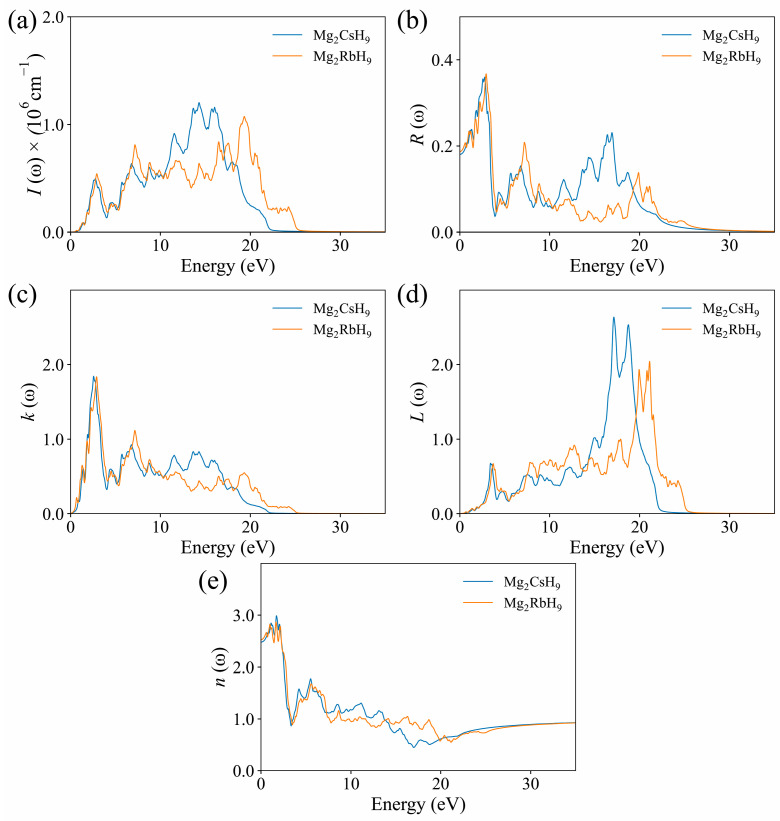
Calculated optical properties of Mg_2_XH_9_: (**a**) absorption coefficient *I*(*ω*); (**b**) reflectivity *R*(*ω*); (**c**) extinction coefficient *K*(*ω*); (**d**) energy loss spectrum *L*(*ω*); and (**e**) refractive index *n*(*ω*).

The absorption coefficients of Mg_2_CsH_9_ and Mg_2_RbH_9_, shown in Figure 6a, were zero in the energy range below their respective band-gaps (0.62 eV and 0.59 eV, respectively), which confirms their semiconducting nature. With increasing photon energy, the absorption peaks were primarily distributed in the UV region (>3.1 eV), indicating a strong tendency of Mg_2_CsH_9_ and Mg_2_RbH_9_ to absorb UV light.

As shown in Figure 6b, the initial reflectivity values of Mg_2_CsH_9_ and Mg_2_RbH_9_ were approximately 0.19 and 0.18, respectively. With increasing photon energy, the maximum reflectivity of Mg_2_CsH_9_ reached approximately 0.36 at 3.2 eV, whereas that of Mg_2_RbH_9_ peaked at approximately 0.38 at 3.4 eV. In the visible light-region, the reflectivity of Mg_2_CsH_9_ was higher than that of Mg_2_RbH_9_; however, in the far-ultraviolet region, Mg_2_RbH_9_ was more reflective. When the photon energy exceeded 27 eV, the reflectivity of both materials approached zero, indicating enhanced transparency to high-energy photons, which is consistent with the attenuation behavior of dielectric responses.

The extinction coefficients *K*(*ω*), presented in Figure 6c, displayed peaks at approximately 1.85 for Mg_2_CsH_9_ and 1.83 for Mg_2_RbH_9_, indicating minor differences between the two hydrides. The similarity in these peak values suggests a comparable degree of interaction between the incident photon radiation and each material. With increasing photon energy, the extinction coefficient showed an overall decreasing trend, indicating a weakened attenuation effect in the high-energy region, which favors light propagation within the material.

The energy-loss function for Mg_2_CsH_9_, presented in Figure 6d, showed a double-peak structure at 16.8 eV (*L* = 2.75) and 18.9 eV (*L* = 2.52), while the loss peaks of Mg_2_RbH_9_ shifted toward the higher-energy region, appearing at 21.2 eV (*L* = 1.92) and 22.8 eV (*L* = 2.08). The overall energy-loss intensity for Mg_2_CsH_9_ was approximately 30% higher than that for Mg_2_RbH_9_, indicating more pronounced collective electron oscillations (plasmons). This may be attributed to the enhanced polarization of the electron cloud induced by the larger radius of the Cs atom. At 0 eV, both substances had a loss function value of 0. The optical and electrical characteristics of these compounds make them suitable for use in LEDs and solar cells.

Figure 6e illustrates the variation of the refractive index *n*(*ω*) of the two hydrides. The static refractive index *n*(0) was approximately 2.5, which meets the requirements for high-refractive-index optical materials (*n* > 2.0). In the range of 7.5–20 eV, the fluctuation amplitude of the refractive index of Mg_2_RbH_9_ (Δ*n* = 0.4) was smaller than that of Mg_2_RbH_9_ (Δ*n* = 0.8), indicating a more stable optical response. When the photon energy exceeded 25 eV, *n*(*ω*) for both materials approached 1, consistent with the dielectric response of a vacuum and confirming the physical mechanism of dielectric polarization loss in the high-energy region.

## 4. Conclusions

Based on first-principles methods, the thermodynamic, hydrogen-storage, mechanical, electronic, and optical properties of magnesium-based alkali metal hydrides Mg_2_XH_9_ (X = Cs, Rb) were predicted. According to the Born stability criteria, both hydrides are mechanically stable. The Poisson’s ratio values confirmed that bonding in both compounds is predominantly ionic. The results also indicate that these alkali metal hydrides exhibit elastic anisotropy. The band structure analyses revealed their semiconducting nature, while the density of state analysis revealed that the *s*-orbitals of hydrogen atoms primarily influence the valence band, whereas the conduction band is mainly contributed by the d-orbital of X atoms, with smaller contributions from Mg atoms. The hydrogen-storage performance analysis revealed that Mg_2_RbH_9_ has a high storage capacity of 6.34 wt%. The optical properties showed that Mg_2_XH_9_ (X = Cs, Rb) exhibit favorable refractive indices, thereby classifying them as high-refractive-index materials. Theoretically, Mg_2_RbH_9_ demonstrates excellent mechanical, electronic, and hydrogen-storage properties, suggesting its strong potential as a candidate for future solid-state hydrogen-storage applications.

## Figures and Tables

**Table 1:** The possible decomposition pathways and relative formation energies *E*_f_ (eV/atom) of these Mg_2_XH_9_ structures.

Structures	Possible Decomposition Path	*E*_f_
Mg_2_CsH_9_	Mg_2_CsH_9_ = 2MgH_2_ + CsH + 2H_2_	−0.118
Mg_2_RbH_9_	Mg_2_RbH_9_ = 2MgH_2_ + RbH + 2H_2_	−0.165

**Table 2:** Optimized lattice constants, volume, formation energies, elastic constants, gravimetric hydrogen capacity, volumetric hydrogen capacity, and desorption temperature of the compounds Mg_2_XH_9_ (X = Cs, Rb).

Parameters	Mg_2_CsH_9_	Mg_2_RbH_9_
a(Å)	8.867	8.663
$V({Å}^{3})$	697.1	650.1
${\Delta H}_{f}(eV/atom)$	−0.387	−0.382
$C_{11}\;(GPa)$	19.6	20.9
$C_{12}\;(GPa)$	10.1	11.1
$C_{44}\;(GPa)$	14.1	14.6
$C_{wt}\%$	4.76	6.34
$\rho_{vol}(gH_{2}/L)$	86.46	92.70
$T_{des}\;\left(K\right)$	286	282

**Table 3:** Calculated bulk modulus, Young’s modulus, shear modulus, elastic anisotropy, *B*/*G* ratio, Poisson’s ratio, Vickers hardness, melting temperature, machinability index, average sound velocity, and Debye temperature for the Mg_2_XH_9_ (X = Cs, Rb) structures.

Parameters	Mg_2_CsH_9_	Mg_2_RbH_9_
B (GPa)	13.3	14.4
E (GPa)	22.3	23.3
G (GPa)	9.1	9.5
A	2.97	2.96
B/G	1.46	1.52
ν	0.22	0.23
$H_{v}\;(GPa)$	1.68	1.56
$T_{m}\;(K)$	613	618
$\mu^{M}$	0.95	0.98
$V_{m}\;(km/s)$	2.49	2.82
$\theta_{D}\;(K)$	303	352

## Data Availability

The original contributions presented in this study are included in the article. Further inquiries can be directed to the corresponding author.

## Appendix A

**Table A1:** Atomic fractional coordinates for Mg_2_XH_9_ (X = Cs, Rb) in the *Pn*$\bar{3}$ phase (Space Group No. 201).

Phases	Pressure (GPa)	Lattice Parameters (Å)	Atoms	Wyckoff Positions (Fractional)		
				x	y	z
*Pn*$\bar{3}$ Mg_2_CsH_9_	0	*a* = 8.8667	Cs (4b)	0.2500	0.2500	0.2500
			Mg (8e)	0.8571	0.8571	0.8571
			H (24h)	0.5302	0.9691	0.7240
			H (12f)	0.7701	0.0000	0.0000
*Pn*$\bar{3}$ Mg_2_RbH_9_	0	*a* = 8.6630	Rb (4b)	0.2500	0.2500	0.2500
			Mg (8e)	0.8552	0.8552	0.8552
			H (24j)	0.5314	0.9686	0.7500
			H (12g)	0.7644	0.0000	–0.0000
